# Low Birthrates and Population

**Published:** 1890-10-18

**Authors:** 


### The Hospital

A Weekly Journal of

Science, Medicine, Nursing, and Philanthropy.

*For Hospitals, Asylums, Medical Practitioners, Students, Nurses, Families, Parishes, Congregations, and the Charitable Public.*

Vol. IX.—No. 212.] [October 18, 1890.

### LOW BIRTHRATES AND POPULATION.

If the historian, the political economist, or the sociologist wishes to know whether a particular nation, or State, or community, has been prosperous during a given period of time, one of the first questions asked is, what has been the rate of increase of its people during the time in question? In general it is assumed that a rapid increase indicates growing strength, and that the slower the growth the less prosperous the community. History tells us that nations and races—like individuals, have their periods of infancy, of growth and development, of decline and decay, that States and governments are transitory things—that the Accads, Assyrians, and Egyptians, the Greeks and the Romans have had their day of power, which has gone to return no more, and that new peoples have been constantly arising to claim the earth and the fulness thereof. Is this the universal law which will govern the future as it seems to have done the past? or are we approaching a period of stable equilibrium in which the earth is to be divided between a few great nations, whose increase in numbers will gradually become less as the countries become more thickly peopled? Few will accept the latter alternative as the most probable, or will be prepared to assert that the governments now existing will be much more permanent than those that have preceded them. Is it then true that a decrease in the fertility of a people indicates that it has entered on the stage of decline? For example, does the diminished birth rate in France warrant the alarm which many of her statisticians and legislators express as to her future when they discuss it?

During the 20 years 1861-1880 the average birth rate in France was 25·9 per 1,000, while during the same period in England and Wales it was 35·3; in Ireland, 26·2; in Italy, 37; in Belgium, 31·8; in Austria, 38·9; in the German Empire, 39. In the year 1888 the birth rate in France was 23·1; in England and Wales, 30·6; in Ireland, 22·9; in Italy, 36·6; in Belgium, 29·1; in Austria, 37·9; in the German Empire, 36·7. It will be seen, therefore, that the birth rates have diminished in all these countries. In Ireland the birth rate has diminished even more than in France. In round numbers the birth rate in Russia is now about double that of France, and although its death rate is also excessive, its annual excess of births over deaths amounts to about 13 per 1,000 of population, while that of France in 1888 was only 1·3, being the lowest, with the exception of Ireland, of any large country of which the figures are known. A few thousand or a few hundred years ago, or even so late as fifty years ago, such facts as these would have had great significance for the future in the eyes of everyone, as they now do in the eyes of most people, but there are many who now think that mere numbers of men are not so important a factor in national life as they were formerly—that wars for conquest in Europe or North America are becoming more and more improbable, and that a country of 38,500,000 people like France may be well satisfied that its increase in population is now slow, since this lessens the risks of overcrowding, and there is no special risk of invasion. With regard to this last point, if we grant that no nation is likely to send an army into France for purposes of forcible conquest, it is still worth noting that the proportion of foreign born in this country increased from 10·6 in 1851 to 21·7 in 1876, and to 30 in 1886 per 1,000 of living population, and that therefore a peaceful invasion is actually going on which may result in a total change in the governing race. But is there an actual progressive, physical deterioration in the French people? Are they losing bodily vigour, becoming more liable to disease and death, less capable of producing healthy offspring? Certainly no one would affirm this from any data which we now have. That there are certain influences tending to produce physical deterioration in the nations of Western Europe is generally admitted. Among these one of the most important factors is the steady increase in the proportion of the population which lives in cities and towns as compared with the increase in the rural districts. It is well known that the conditions of town life are much more destructive to human health and life than are those of the country; that few families of the commercial and working classes last more than three generations in a city unless crossed with fresh importations from without, and that the steady stream of people from the country to the city, brought about by the conditions of modern life, and especially by the development of steam power and electricity, is a necessity not only for the increase, but for the maintenance, of the urban populations. In France, however, this factor is not of so much importance as it is in England, nor is emigration draining the country of its most energetic, vigorous, and intelligent youth to the extent that it is doing in England, Germany, and Scandinavia. The influence which is producing diminished birth rates in France appears to be very largely the will of the people themselves. Arsene Dumont, one of the latest writers on this subject, says: "If natality is diminishing amongst us, if each married couple in France has an average of not more than three infants born, as against four in England and five in Prussia, it is because the French husband and wife do not wish to have a larger number. On this point the national prudence finds its formula, not in the advice, unknown to the masses, of certain writers on political economy, but in popular aphorisms, of which one of the best known is, that as regards children in a family, two are better than twelve." As shown above, the diminution in the birth-rate has been even greater in Ireland than in France, but the cause of this is probably the emigration of large numbers of girls and young women rather than voluntary abstinence. This policy of deliberately restricting the number of offspring is pursued in all countries by a certain number of families, but in France the practice is more general than elsewhere, and has a more evident effect upon the national increase.

Is this state of affairs an evil which should be combated? Dumont states his ideal for France from the demographic point of view as being that it should have a birth-rate of 25, a marriage rate of 8, and a death-rate of 15 per 1,000 of population. This would give about three children to each marriage, and an annual increase of the whole population to the extent of 1 per cent. In other words, the change which he would make in the existing state of things would be mainly by reducing the death-rate from about 22 to 15 per 1,000, and he would only increase the existing birth-rate about 2 per 1,000—*i.e.*, from 23 to 25. With regard to the desirability of the reducing sickness and death rates as much as possible there is no dispute, the controversy is always with regard to the merits of any particular method that may be proposed for effecting this, and whether its results will be worth its cost. France is doing much to educate the people in sanitary matters, but the practical application of the approved methods of securing pure water, good drainage, &c., is being made very slowly. Even Paris has much need of immediate improvement in this direction, and we fear it will be a long time before her death-rate will be brought down to 20 per 1,000 annually, although the means of doing this are almost certainly known.

In a paper recently presented to the Academy of Medicine, M. Gustave Lagneau (see "Bulletin de L'Académie de Médicine," June 24) treats on measures to prevent decrease of population in France. He shows that marriage is becoming less and less frequent, and that it is deferred to the later ages. The mean age as to marriage appears to be 29 years and 9 months for males, and 25 years for females. If we exclude widowers and widows the mean age at which marriage occurs for those who have never been married is for males 28 years, and for females 23 years and 5 months. In England the mean age at marriage for all males is 28·2, for all women 25·9; while for those who have never been married it is 26·2 for males, and 24·7 for females. With late marriages there come tendencies to alcoholism, to prostitution, to illegitimate natality, and bad habits of various kinds, which tend either to produce sterility, or to greatly increase the death-rate among infants. The slow increase of population in France has nothing to do with the quantity of food which the country is producing, or is capable of producing, from its own soil, for it is the quantity of sustenance which a nation is able to procure, either from its own soil, or by importation, which limits its increase; in other words, the amount of wealth which it is capable of producing. It is from this point of view that increase of numbers is considered to be a help to a nation in the acquisition of wealth, since one pair of hands, if allowed fair play, can more than satisfy the demands of one stomach.

In old countries where there is no great amount of new land awaiting to be taken into cultivation, the increase of any class of the people is usually in inverse proportion to the amount of sustenance which it can easily command. That is, the poor increase most rapidly, the middle classes more slowly, and the upper, or wealthy, classes not at all. Attempts to increase the birth rate in a country like France would probably be useless, and it is by no means certain that it is desirable that it should be increased. The melancholy forebodings of loss of national power and prestige, threatened by the low natality of the country, which some modern French statisticians have expressed, do not seem to be justified in the present state of the civilized world. When man is viewed solely as a fighting animal it may be proper to have anxieties about the supply available for army purposes, but if he is considered as a producer of food, clothing, habitations, objects of art, etc., in short of the means of sustaining life and of producing happiness, it is by no means clear that increase in numbers beyond a certain point is in itself desirable, or worth making special effort to provide for. Finally, a diminution in the birth rate of a country is not necessarily an evil in itself. It is a sign of a change in the people either as to habits or as to age distribution, especially as connected with a lessening of the proportion of women of from 15 to 45 years of age present in the population, and from this last point of view the results of the census of 1891 in France and Ireland will be of great interest.

